# Hidden hearing loss is associated with loss of ribbon synapses of cochlea inner hair cells

**DOI:** 10.1042/BSR20201637

**Published:** 2021-04-09

**Authors:** Feng Song, Bin Gan, Na Wang, Zhe Wang, An-ting Xu

**Affiliations:** 1 Department of Otolaryngology, Lanling County People’s Hospital, Lanling, Shandong 277700, P.R. China; 2 Department of Otolaryngology, The Second hospital, Cheeloo College of Medicine, Shandong University, Jinan, Shandong, 250033, P.R. China; 3 Translational Medicine Core Facility of Advanced Medical Research Institute, Shandong University, Jinan, Shandong 250100, P.R. China; 4 NHC, Key Laboratory of Otorhinolaryngology, Shandong University, Jinan, Shandong 250100, P.R. China

**Keywords:** auditory brainstem response, inner hair cells, Moderate-intensity noise, noise-induced hearing loss, ribbon synapse

## Abstract

The present study aimed to observe the changes in the cochlea ribbon synapses after repeated exposure to moderate-to-high intensity noise. Guinea pigs received 95 dB SPL white noise exposure 4 h a day for consecutive 7 days (we regarded it a medium-term and moderate-intensity noise, or MTMI noise). Animals were divided into four groups: Control, 1DPN (1-day post noise), 1WPN (1-week post noise), and 1MPN (1-month post noise). Auditory function analysis by auditory brainstem response (ABR) and compound action potential (CAP) recordings, as well as ribbon synapse morphological analyses by immunohistochemistry (Ctbp2 and PSD95 staining) were performed 1 day, 1 week, and 1 month after noise exposure. After MTMI noise exposure, the amplitudes of ABR I and III waves were suppressed. The CAP threshold was elevated, and CAP amplitude was reduced in the 1DPN group. No apparent changes in hair cell shape, arrangement, or number were observed, but the number of ribbon synapse was reduced. The 1WPN and 1MPN groups showed that part of ABR and CAP changes recovered, as well as the synapse number. The defects in cochlea auditory function and synapse changes were observed mainly in the high-frequency region. Together, repeated exposure in MTMI noise can cause hidden hearing loss (HHL), which is partially reversible after leaving the noise environment; and MTMI noise-induced HHL is associated with inner hair cell ribbon synapses.

## Introduction

Hair cell loss and damage are important reasons for noise-induced hearing loss (NIHL), which may affect the auditory function after noise exposure [1]. High-intensity noise exposure can cause temporary threshold shift (TTS); it also destroys the ribbon synapses between the inner hair cells (IHC) and type I afferent nerve fibers. Cochlear coding deficits can occur after transient noise exposure without permanent changes in sensitivity [2]. This is also called hidden hearing loss (HHL) or silent coding deficits, when tangible cochlea is impaired without changes in sensitivity [3,4]. Increasing data suggested that noise-induced synaptopathy may underlie the HHL phenomenon [5,6]. In guinea pig studies, low doses of intensity noise exposure can sharply damage the ribbon synapses between IHCs and spiral ganglion neurons (SGNs) [7]. Generally, damage and loss in both pre- and post-synaptic structures (as identified by staining on ribbons and post synaptic density (PSD)) can start up the repair process. Cochlear synaptopathy is a loss of, or damage to, synaptic contacts between cochlear hair cells and auditory nerve fibers (ANFs). However, the dynamic mechanism of IHC repair and the influence of cochlear synapses reduction in the audiology is not fully clear. Besides, no studies have examined the effects of medium/long-term medium or moderate intensity noise exposure on the cochlea. Here, we applied intermittently medium-intensity noise to expose guinea pigs for a week and noticed significant changes in auditory brainstem response (ABR) wave-I and wave-III and compound action potential (CAP) responses. This work found that, after exposure to medium-term and moderate-intensity (MTMI) noise, NIHHL can be developed, and changes of ABR waves and CAP amplitudes could be early markers of cochlear synaptopathy.

## Materials and methods

### Animals and procedures

Male albino adult (280 g) guinea pigs were purchased from Shanghai JieSiJie Laboratory Animal Co. Ltd (Shanghai, China). All animals were approved after Preyer reflex test, otoscopic examination, and the normal hearing thresholds were determined with tone burst-evoked ABR examination. Animal procedures were approved by the Committee for Laboratory Animals of Shandong University (KYLL-2015 GJ, P-0036).

Thirty-eight guinea pigs were divided into four groups (one control group, *n*=10, and three groups for noise exposure, *n*=8, 8 and 12, respectively). After the basal ABR examination, the noise exposed groups were administrated with 1 week of broadband noise at 95 dB SPL (4 h/day). ABR and CAP were tested again 1 day, 1 week, and 1 month post-noise exposure, respectively, and three groups were divided accordingly (the 1DPN group: 1-day post noise exposure, 1 WPN: 1-week post noise exposure, and 1 MPN: 1-month post noise exposure). After auditory function tests, guinea pigs were initially anesthetized by an intraperitoneal injection of 5% chloral hydrate solution (350 mg/kg.BW). Then, they were killed by dislocation of cervical vertebra. The animal work has taken place in Shandong University.

### Noise exposure

The broadband noise was set at 95 dB SPL for a continuous 4 h, as previously reported [8]. Animals were placed 50 cm below two loudspeakers (Tucker-Davis Technologies, U.S.A.) in a cage. The noise intensity was monitored by a sound level meter (RION NL-11A, Japan) to ensure animals received the expectant dosage with errors less than 1 dB. During the entire process, animals were awake and free to move, with sufficient food and water.

### Auditory function tests

The ABR threshold measurement and CAP response measurement were two commonly used tests for cochlear function assessment [9–12]. Animals under ABR and CAP tests were anesthetized with pentobarbital sodium (30 m/kg) and maintained body temperature at 37.5–38.0°C. For each animal, the head was fixed in a head holder, the left middle ear bulla was opened, and image of the cochlear apex was observed. The stimulation signal generation and evoked potential recordings were administrated by TDT system III (Alachua, FL, U.S.A.). The sounder was placed 10 cm in front of the ears. To record ABRs, the scalp needle electrode was inserted into the skin under local anesthesia for recording, the subcutaneous positive input electrode was located in the center top of the head, the reference electrode and the ground electrode were located behind the left ear and right ear, respectively. For CAP recording, we applied sound stimuli of alternating polarity from (10–80 dB SPL, 600 Hz to 32 kHz). A silver electrode was fixed on the round window membrane and penetrated on the bulla inferior and posterior to the external ear canal.

The evoked responses were amplified 20-fold with a TDT pre-amplifier (RA16PA, sampling rate 25 kHz). The responses were averaged 1000-times for ABR and 100-times for CAP. ABR thresholds were measured across the frequencies from 1 to 32 kHz with tone bursts at the rate of 21.1/s. At each frequency, the test was performed in a degressive sequence, starting from 90 dB SPL, and weakened in 5-dB steps until the ABR response disappeared. The threshold was determined as the lowest level at which a repeatable wave III response could be observed. The representative ABR trace was demonstrated in Supplementary Figure S1.

The detailed acoustic stimulus designed for recording ABR, ABR with masking, CAP and amplitude modulation CAP (AM-CAP) were as follows. For ABRs stimuli: tone bursts of 10-ms duration with cos2 gating, 0.5-ms rise/fall time. The signal was pre-amplified by 20×, proceeded with a bandpass (100–3000 Hz) filter, and superimposed 1000-times. It started with 90 dB SPL and decreased by 5 dB steps. The lowest sound intensity of the wave-III was documented as the ABR threshold. Next a combination of 90-dB SPL clicks with uniformly increasing broadband noise (as masking noise, started at 30 dB and increased at 10-dB steps to 80 dB SPL) was given. The amplitude and latency of wave-I and wave-III were documented. The amplitude was defined as the difference between the current peak value and the immediately following valley value.

The CAP and AM-CAP were recorded to evaluate auditory temporal processing ability. CAPs were recorded in response to clicks or tone bursts across the same range of SPLs as used in ABR recording. Compared with ABR, the tone bursts were designed at different frequencies (1, 2, 4, 10, 20, 24 and 32 kHz). Both CAP thresholds and peak amplitudes were measured. The peak-to-peak value was regarded as the CAP peak amplitude. For AM signals, the carrier frequency was designed at the frequencies 2, 10 and 20 kHz), and the modulation frequencies varied between 93 and 996 Hz, while the carrier strength was fixed at 80 dB SPL, and the modulation depths varied from 10 to 100% (10, 20, 40, 60, 80 and 100%). Responses were recorded continuously and averaged by looping into a 700-ms window. The response amplitudes at modulation frequencies were calculated and expressed in dB using Fast Fourier Transform (FFT) with in the TDT BioSig software (8192 points). Envelope following responses (EFRs) were considered positive if a clear peak in FFT was identified at 93 or 996 Hz with amplitude 3 dB higher than the troughs above and below the peak.

### Morphology analysis

After killing, ears from each animal were used for immunostaining against ribbons and PSD. To observe the ribbon synapse structure, the cochlea was fixed with 4% paraformaldehyde for 24 h, the bone wall, the spiral ligament, the vestibular membrane, and the tectorial membrane were removed under the dissecting microscope. Afterwards, the cochlea was permeabilized with 1% Triton X-100 in PBS for 45 min, incubated for 30 min in 10% goat serum in PBS and then incubated in the mixture of two primary antibodies (rabbit anti-CtBP2 C-terminal-binding protein 2, Abcam, cat. #ab128871, 1:200, and mouse anti-PSD95, Abcam, cat. # ab2723, 1:200 in the primary antibody diluent) overnight. After 3 times of wash (10 min each), each cochlea was inculcated with secondary antibodies (goat anti rabbit IgG, Abcam, cat.# ab175471, 1:200, and goat anti mouse IgG2a, Invitrogen, 1:200, A21131) for 1 h at room temperature. Samples were washed three times with PBS, and then immersed in 10% EDTA. The basilar membrane was dissected into four pieces according to the corresponding cochlea turn. Next, the basilar membrane was mounted on microscope slides. Confocal z-stacks from each ear were obtained using a high-resolution oil-immersion objective (×100) on a confocal laser-scanning microscope (Zeiss LSM 780). Image stacks were then ported to the image-processing software (Zen 2010 blue and ImageJ), with 0.1 μm layer distance, and the confocal z-stacks were reimaged for 3-D reconstructions. The locations in terms of lengths or distances from the apex were mapped [13–15]. A total of 17 regions were analyzed across the basilar membrane, and in each cochlear region, the synapse number was calculated. The final puncta for elements staining were obtained by calculating the average number of pre- or postsynaptic elements for each IHC.

### Statistical analysis

Data were analyzed using IBM SPSS 22.0 and GraphPad Prism 6. The quantitative data were expressed as mean ± standard error, and the differences in ABR or CAP responses among groups were analyzed using Tukey’s multiple comparisons in the two-way ANOVA method. The number of ribbon synapses among groups was analyzed using Tukey’s multiple comparison test in one-way ANOVA. A *P*-value less than 0.05 was considered statistically significant.

## Results

### MTMI noise impaired auditory function 1-day to 1-month post exposure

**Figure 1:**
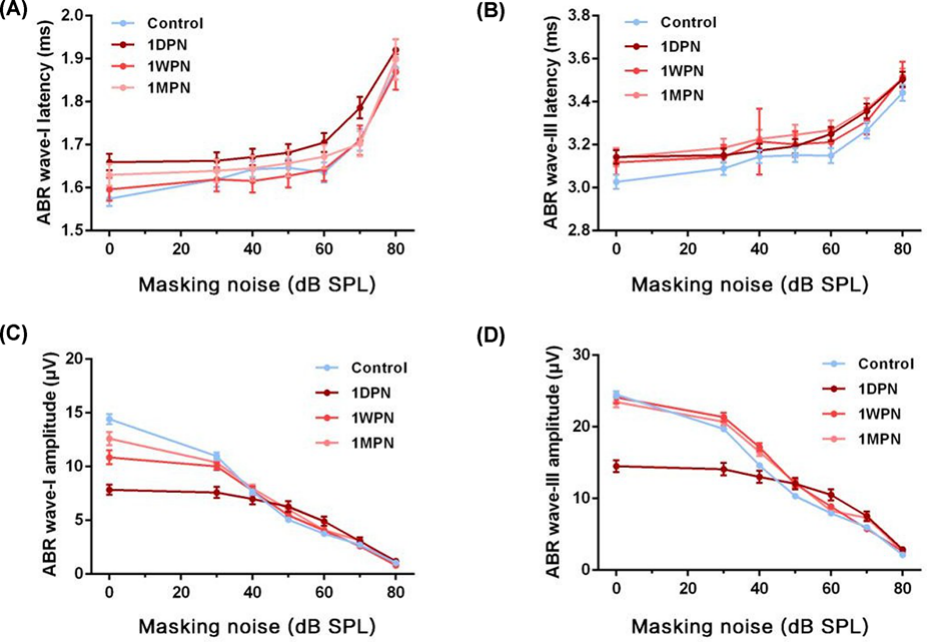
MTMI noise evoked changes in ABR under masking noise (**A**) ABR wave-I latency under different intensities of masking noise. (**B**) ABR wave-III latency under different intensities of noise background. (**C**) ABR wave-I amplitude under different intensities of masking noise. (**D**) ABR wave-III amplitude under different intensities of masking noise.

First, ABR examination was conducted in masking noise environment to evaluate the impact of MTMI noise. For ABR wave-I and III latencies, no significant differences were found among groups (Figure 1A,B). The amplitudes of ABR wave-I and wave-III decreased 1 day after noise exposure and gradually restored during 1 week/month of recovery (wave-I: masking factor: F = 10.20, *P*<0.01, group factor: F = 306.5, *P*=0.0001; wave-III: masking factor: F = 19.11, *P*<0.01; group factor: F = 391.4, *P*<0.01) (Figure 1C,D). Collectively, MTMI noise significantly impairs auditory function assessed by ABR 1 day later, and the residual effect may last for at least 1 month (Figure 1A,B).

**Figure 2:**
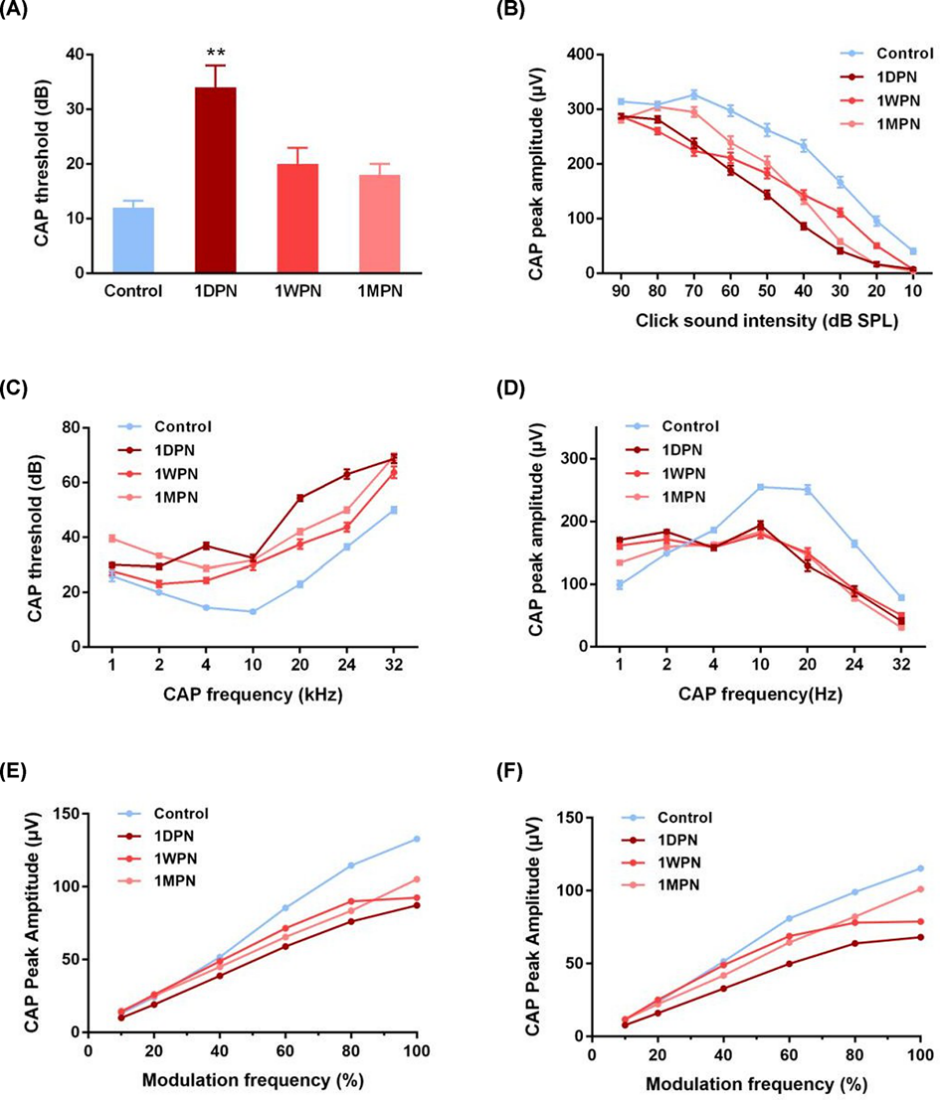
MTMI noise caused changes in CAP responses (**A**) Under the 90-dB SPL click acoustic stimulation, the 1DPN group showed an increased CAP threshold, and this parameter recovered in the 1WPN and 1MPN groups. (**B**) CAP peak-to-peak amplitudes under different click stimulations in the noise exposed groups. (**C**) Under the tone burst acoustic stimulation, CAP thresholds were elevated in the noise exposed groups at different frequencies. (**D**) Under the tone burst acoustic stimulation, CAP peak amplitudes were suppressed in the noise exposed groups at different frequencies. (**E,F**) AM-CAP responses were suppressed in different frequency modulation depths. (E) 20 kHZ carrier at 80 dB SPL strength, coupled with a modulation frequency of 996 Hz. (F) On a 60 dB SPL white noise exposure backgrounds (NEBs). (Control: *n*=10, 1DPN: *n*=8, 1WPN: *n*=8; 1MPN: 12).

The impact of noise exposure on cochlear function was evaluated by the CAP responses to two types of stimuli: click and tone burst. For the click triggered responses, the CAP threshold of the 1DPN group was significantly elevated (*P*<0.05 vs control), and no statistically significant differences were found in the other two noise exposed groups (Figure 2A). Still, the mean CAP thresholds were slightly higher than control. Similarly, the CAP peak amplitudes were decreased in three noise suffered groups compared with the control group (click sound intensity factor: F = 450.6, *P*<0.0001; group factor: F = 116.1, *P*<0.0001) (Figure 2B). This finding strongly implied that the auditory function had not acquired a full repair in the following month. On the other side, curves of CAP threshold and amplitude triggered by the tone burst were drawn (Figure 2C,D). The CAP threshold increased with tone burst frequencies, and three noise exposed groups showed significantly shift-up curves compared with the control curve (*P*<0.05 each noise exposed group vs control) (Figure 2C). Similar to the data of click stimuli, CAP amplitudes corresponding to high frequencies were suppressed (Figure 2D). However, in the low-frequency range, the CAP peak amplitudes were enhanced. This interesting finding may be due to a sensitized disorder towards low-frequency signals after noise exposure. Next, AM-CAP responses were recorded as a support. For all animals, the peak amplitude increased with modulation frequencies, and three noise exposed groups had significantly decreased amplitudes in both responses to different frequency modulation depths (Figure 2E) and in 60 dB SPL white noise exposure backgrounds (Figure 2F) (*P*<0.05 each noise exposed group vs control). No significant differences were found among three noise-exposed groups. These findings strongly highlight an incomplete repair of AM-CAP peak amplitude even one month after MTMI noise exposure.

### Noise-induced cochlear synaptopathy

**Figure 3:**
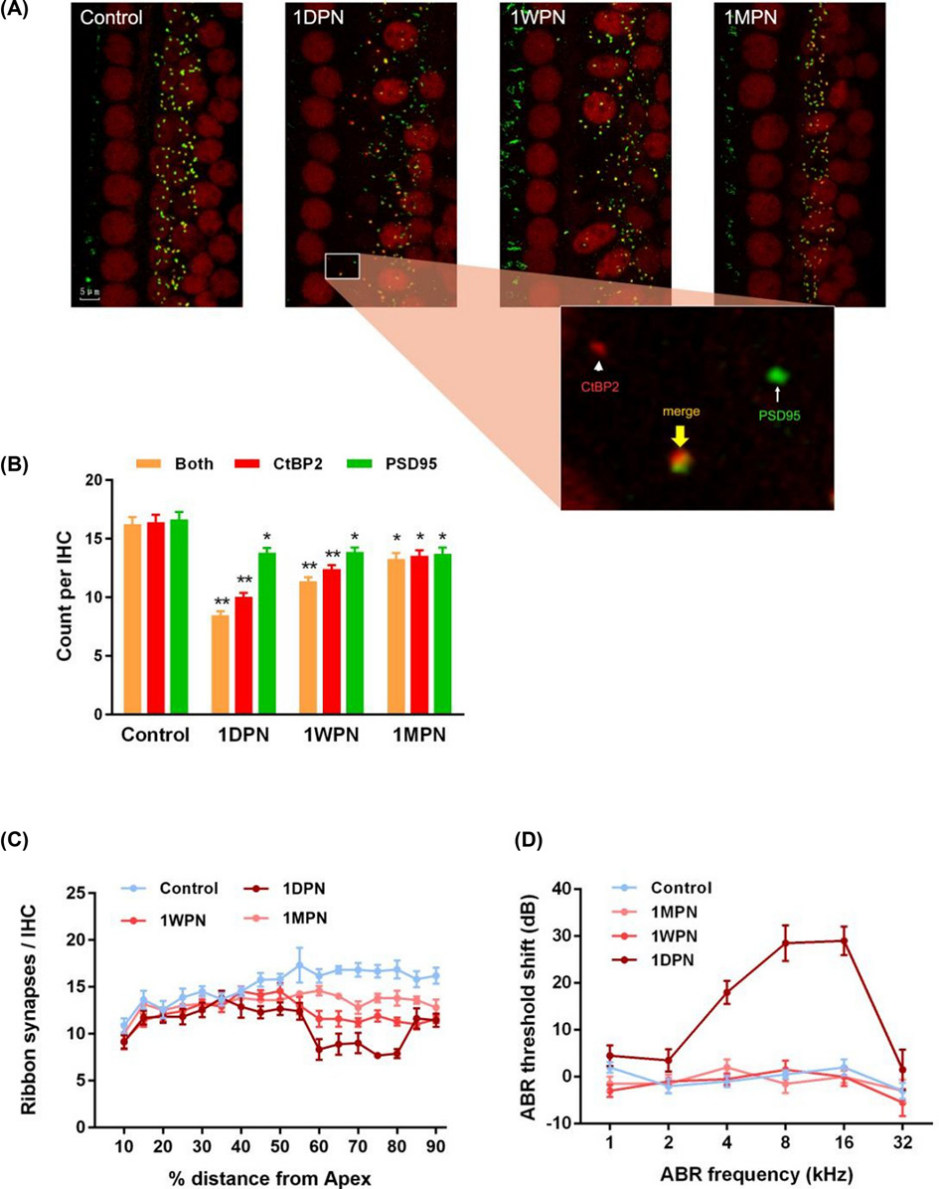
MTMI noise caused cochlear synaptic changes in morphology and function (**A**) Ribbons (stained red against CtBP2) and PSD95 (green) in different groups. The paired synaptic structure was indicated by the yellow arrow and double stained (yellow). (**B**) Noise-induced decreases in synaptic components (numbers per inner hair cell, IHC) in the region corresponding to 5000–16000 kHz frequencie in exposed groups. (**C**) Noise-induced changes of paired ribbon synapse (counts per IHC) along the whole cochleae, with a clear loss in the high-frequency regions (55–85% distance from Apex). (**D**) The ABR threshold shift at different frequencies revealed that the 1DPN group had a serious threshold shift at high frequencies, which was consistent with the cochlear synaptopathy changes at the high-frequency regions. *P<0.05 vs control, **P<0.01 vs control.

To reveal the impairment of cochlear synapses induced by noise, immunofluorescence staining was used to observe the dynamic changes in cochlear synapses at different time points after the noise exposure. The presynaptic ribbons were immune-stained with an anti-CtBP2 antibody, and the postsynaptic terminals were stained with an antibody to PSD-95, the specific PDZ-domain protein (Figure 3A). No apparent changes in hair cell shape, cell arrangement or cell number were observed. At the synaptic level, both presynaptic ribbons and postsynaptic terminals were reduced after noise exposure (Figure 3B), and this change was most significant in the 1-day group (*P*<0.01). The amount of CtBP2 and PSD-95 showed a partial recovery in the following 1 week or 1 month (but still lower than the control group). Moreover, as reported previously, the co-stained (paired) synapses were markedly reduced at 1DPN (F = 56.91, *P*<0.01; paired synapse: F = 46.34, *P*<0.01; ribbon: F = 4.075, *P*=0.011; PSD: F = 31.24, *P*<0.01). This result confirmed a damage on cochlear synapse from noise. In particular for the 1DPN group, an overall loss of 48.9% paired synapses was found, with 38.8% reduction in presynaptic ribbons and 17.1% reduction in postsynaptic terminals. It is interesting that no difference was found in the PSD amount among three noise exposed groups (*P*>0.05), which implied that the postsynaptic injury induced by noise had extremely limited recovery in the following one month. The impairment of cochlear synapse showed a regional feature, as Figure 3C shown. The decrease was mainly in the region of 60–80% distance from apex, but not the overall length, which coincided with the changed range of corresponding threshold shift (2000–20000 Hz, Figure 3D). Although there is a mismatch between CAP and ABR thresholds, this may be due to different sensitivity of two tests (especially towards the 1DPN group). Also, the scattered distribution of the ribbons was changed after noise exposure. Some ribbons were located on the side of the modiolus, but not close to the outer hair cells, which was consistent with a recent work by Boero et al. revealed that the α9α10 nAChR complexes on outer hair cells may be involved in age-related hearing loss [16]. However, this trend is not fully significant. Together, MTMI noise may cause clear pathological morphology changes in presynaptic ribbon synapse and postsynaptic terminals, which coincide with the auditory function defects; and the pathological alteration was difficult to recover at least within 1 month.

## Discussion

The major findings of the present study are: (1) repeated exposed in MTMI noise may cause hearing loss within 1 month, which is reversible after leaving the noise environment; (2) MTMI noise exposure may damage IHC ribbon synapses. Before the present study, there are very few works focusing on the effects of MTMI noise on cochlear synaptopathy and function defects. The significance of the present study is that we provided further evidence supporting cochlear synaptopathy may be involved in HHL.

It has been widely accepted that noise may induced damage on IHCs and SGNs in the cochlea [17], including synaptopathy, which is one of the main reasons of HHL. Increasing evidence has shown that exposure to even a single dose of high-intensity noise can generate a substantial degree of cochlear synaptopathy [2,18]. A previous study revealed that coding deficits in noise induced HHL may stem from incomplete repair of ribbon synapses in the cochlea [2]. Single acoustic overexposures (especially low intensity) usually cause transient threshold elevation and also irreversible damage of the synapses between cochlear nerve terminals and IHCs, and sometimes induces permanent threshold shifts (PTS). However, the impact of medium/long-term low/medium-intensity noise is not fully clear, and for MTMI noise exposure, few studies have observed the corresponding morphology changes (especially focusing on both pre-synaptic ribbons and post-synaptic terminals).

In animal studies showing the temporary elevation in hearing thresholds caused by noise exposure, there may be reduced neural responses in an adaptive way, and theoretically the HHL-like state quickly recovers. It has been known that after a single, brief noise exposure, the damaged and the totally destroyed synapses can be partially repaired, but the repaired synapses are functionally abnormal [17]. The mechanism underlying noise induced threshold elevation and hearing recovery is closely related to IHC functions, especially cochlear synapse plasticity. Kujawa and Liberman showed that 2 h of 100 dB SPL noise (8–16 kHz) exposure killed half of IHC/auditory nerve synapses in high-frequency regions permanently but sensitivity to quiet sounds was easy to recover [6]. Although their research focused on the acute noise stress, the conclusion was consistent with our findings. Loss or recovery of both ribbon and synapse plays a crucial role in the final auditory outcomes, but we believe that cochlear synaptopathy is a more direct reason of HHL, under noise exposure or during aging process [19]. Liberman et al. [32] suggested that age-related decline in spiral ganglion cells may contribute to function loss of hearing-in-noise, especially the ganglion cell survival in the high-frequency segments of cochlear location (which was similar to our result Figure 3D) [19].

Based on the ABR and CAP results in our work, repeated MTMI noise harm responses to the high-frequency stimuli but not low-frequency, which is similar to dysacusis in the elderly. Synapses between cochlear nerve terminals and IHCs are the most vulnerable elements in both noise-induced and age-related hearing loss [20]. Mouse experiment suggested that synaptic connections between cochlear neurons and hair cells decreases by 50% over lifespan and 25% at middle age, when there is not yet any loss of hair cells [21]. Liberman et al. pointed that synaptic elements nearly lost immediately after the 2-h noise exposure, that there is a reversible down-regulation of GluR expression in the peripheral terminals [3]. Therefore, cochlear synapse loss may be a more direct factor that impacts HHL and its recovery. In human studies, supportive evidence also exists. Liberman recently showed that in the aging human cochlea there is primary neural degeneration which might explain a loss of speech discrimination ability in the elderly [22]. Moreover, another explain of this finding is the sound-evoked feedback reduction of cochlear amplification [23]. When associated with sustained discharge rate, the threshold shift might be protective during noise exposure. Moreover, we noticed that CAP amplitudes corresponding to high frequencies were suppressed but in the low-frequency range the CAP peak amplitudes were enhanced. This may be due to a sensitized disorder towards low-frequency signals. Although the detailed mechanism is not clear, it is possibly a homeostasis as a protection mechanism. Another explanation is that disordered CAP response is an overall left shift of the CAP peak curve, that the max amplitude in the response to medium frequency slightly turned to the left range (low frequency). However, the deep mechanism of this finding requires further study.

We observed that the MTMI noise exposure inhibited the responses to high frequencies and caused synaptopathy mainly in the region of 60–80% from the apex. This region is related to the input of high-frequency signals [24,25]. The region near apex had similar CtBP2 and PSD-95 distribution and expression levels (not shown). Collectively, auditory function tests and morphology analysis can mutually support, which both highlighted that MTMI noise induced defects toward high-frequency signals are possibly due to damage in the synapses located at the 60–80% cochlear region from the apex.

It is worth mentioning that decreased output from the cochlea may trigger a compensatory neural gain in the auditory brainstem (reported in ABR wave-III) [26,27]. Years ago, Hickox and Liberman claimed that noise-exposed mice with cochlear neuropathy show hypersensitivity to sound, which suggests a link between AN damage and hyperacusis [28]. Similarly, we also observed an enhanced CAP amplitude under low frequencies (lower than 4 kHz) noise exposure (Figure 2D). Another explain is that, after HHL, it is easy to cause tinnitus, and the stochastic resonance plays a role during the period of hearing threshold recovery [29]. Together, MTMI noise may lead to complicated disorders in cochlear functions, majorly exhibiting impairment in high frequencies and enhancement in low frequencies.

Still, the present study has some limitations. Generally, abnormalities in wave-I and wave-II indicate lesions of the distal auditory nerve, and abnormalities in waves III–V indicate intracranial brainstem lesions [30]. In particular, ABR wave-I amplitude has a close relationship with physiological and perceptual consequences of noise exposure [30]. Therefore, we mainly focused on ABR wave-I and III. However, observed alterations on SGN implies that it has a reason to pay attention on wave II [31]. Our further work will investigate the ABR wave-II changes. Besides, the novelty of the present study largely depends on the use of MTMI noise, but there are limited findings regarding the deep mechanisms of loss of ribbon synapses of cochlea inner hair cells. Additionally, we have not fully told the functional deficits associated with the synaptic damage/loss (but without PTSs), the current functional changes are mainly supported by ABR-wave and CAP-amplitude.

In summary, the present study revealed that loss/recovery of the ribbon synapses in the cochlea is associated with ABR-wave and CAP-amplitude changes in NIHHL developed by MTMI noise. These alterations might be associated with cochlear synaptopathy.

## Supplementary Material

Supplementary Figure S1

## Data Availability

The datasets generated and/or analyzed during the current study are available from the corresponding author on reasonable request.

## Abbreviations

1DPN: 1-day post noise
1MPN: 1-month post noise
1WPN: 1-week post noise
ABR: auditory brainstem response
AM-CAP: amplitude modulation compound action potential
BW: Body Weight
CAP: compound action potential
FFT: Fast Fourier Transform
HHL: hidden hearing loss
IHC: inner hair cell
PSD: post synaptic density
PTS: permanent threshold shift
SGN: spiral ganglion neuron
SPL: Sound Pressure Level
