# Supplementary figures and images for: Hidden hearing loss is associated with loss of ribbon synapses of cochlea inner hair cells
Source: Biosci Rep. 2021 Apr 9;41(4):BSR20201637. doi: 10.1042/BSR20201637 (PMC8035623; doi:10.1042/BSR20201637)

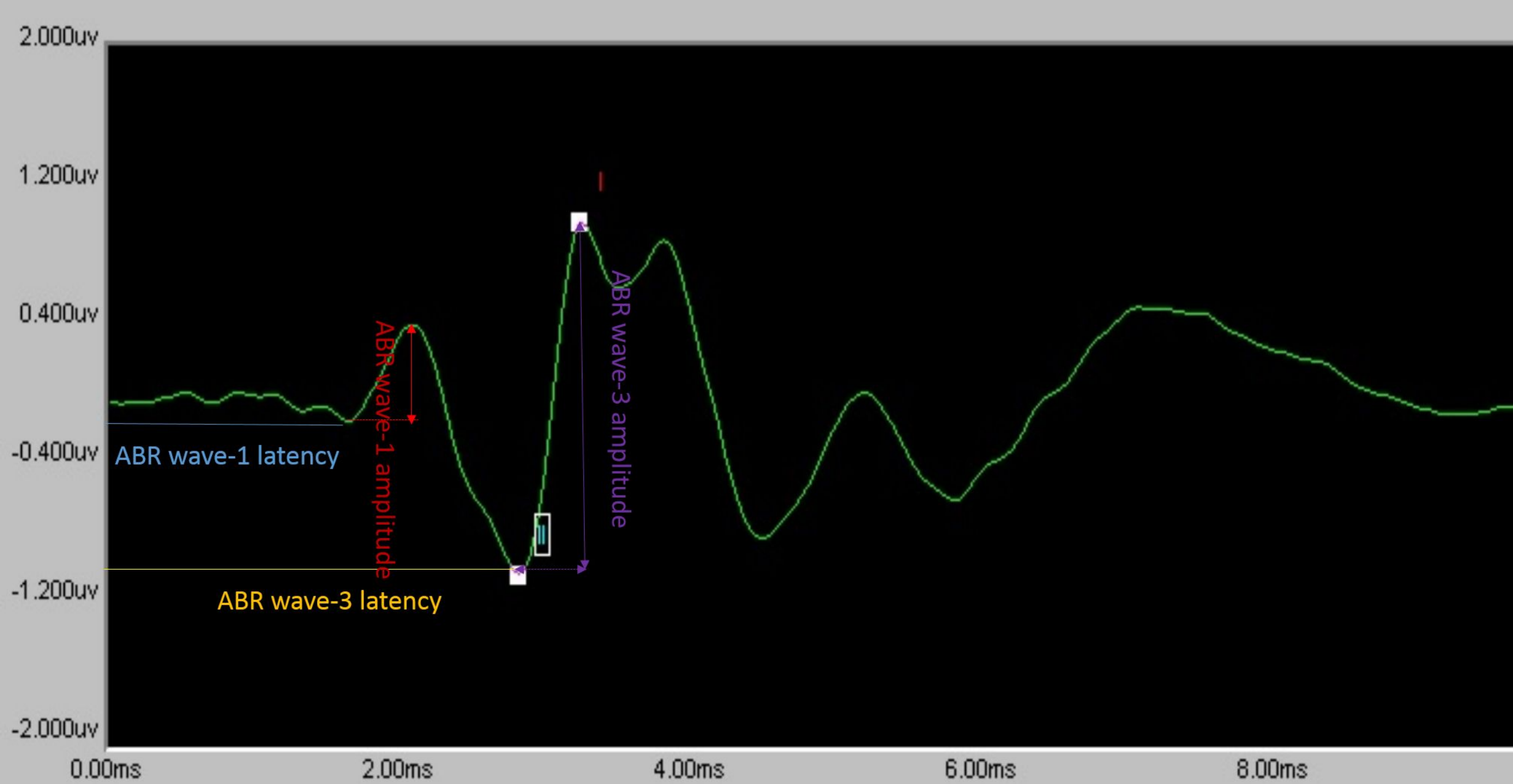

Supplement: Supplementary Figure S1 [file BSR-2020-1637_supp.pdf]
